# Overexpression of AdeIJK RND efflux pump in *Acinetobacter baumannii* using the mini-Tn***7***T-based single copy induction system can lead to *lacI^q^* mutation

**DOI:** 10.1128/spectrum.01609-25

**Published:** 2025-11-10

**Authors:** Ruwani L. Wimalasekara, Rakesh Patidar, Dawn White, Ayush Kumar

**Affiliations:** 1 Department of Microbiology, University of Manitoba 8664, Winnipeg, Manitoba, Canada; University of Saskatchewan, Saskatoon, Saskatchewan, Canada

**Keywords:** gene exrepression, efflux pumps

## Abstract

Chromosomal gene complementation with inducible expression is a powerful approach for investigating bacterial gene function. The pUC18T-mini-Tn*7*T system enables isopropyl β-D-1-thiogalactopyranoside (IPTG)-inducible, site-specific genomic integration of genes in single copy via the P*tac-lacI*^q^ regulatory system. While widely used, limitations remain underexplored. Here, we report a rare and possibly protein-specific but critical caveat of this system during overexpression of AdeIJK, a resistance-nodulation-division efflux pump, in *Acinetobacter baumannii*. IPTG exposure led to the emergence of IPTG-unresponsive mutants with the loss of IPTG-dependent gene expression. Sequencing identified a shared R197S mutation in *lacI*, impairing IPTG binding and derepression. These findings underscore the need for vigilant monitoring of regulatory elements when using inducible systems, particularly during the expression of genes whose overexpression is not well tolerated by bacteria.

**IMPORTANCE:**

Genetic tools are essential for gaining deeper insights into bacterial systems. Though these tools have been of great value in the study of genotype-phenotype relationships, it is equally important to recognize their limitations. We believe this observation will help raise awareness of a potential constraint associated with the widely used mini-Tn7 gene expression system and enable researchers to avoid misinterpretation of experimental results. Being conscious of this limitation, subtle changes that may have otherwise gone unnoticed can be more effectively identified.

## OBSERVATION

Gene complementation with controllable expression is the gold standard for establishing gene function in bacteria (1). Two principal strategies are commonly employed: introduction of a plasmid or chromosomal integration. Plasmid-based systems are easy to use but are often limited in controlling gene expression due to varying plasmid copy number (2). Conversely, chromosomal integration allows for more precise regulation of gene expression by introducing a single copy of the gene into a defined genomic locus. Additionally, chromosomal insertions are genetically stable and circumvent the requirement for continuous antibiotic selection, which is often necessary with plasmid-based systems (2, 3).

The pUC18T-mini-Tn*7*T vector system is a versatile tool for the chromosomal integration of single genes or small operons into the genome of bacteria, facilitating site-specific genomic insertion into a neutral *att*Tn*7* site and enabling controlled gene expression through the use of the P*tac-lacI^q^* regulatory system. In this system, *lacI^q^* encodes a repressor protein (LacI) that binds to the *lac* operator sequence located downstream of the P*tac* promoter, thereby blocking transcription. An exogenously added inducer, isopropyl β-D-1-thiogalactopyranoside (IPTG), can bind with LacI, resulting in a conformational change within the protein that releases the repressor from the operator region and permits transcription of the gene(s) of interest. Thus, this system allows IPTG-dependent, titratable gene expression, which is advantageous when investigating the physiological impact of gene expression levels and protein overproduction in bacteria (3). The pUC18T-mini-Tn*7*T vector has been used successfully to study antibiotic tolerance, stress responses, virulence during infections, and reporter gene expression in important pathogenic variants of *Pseudomonas aeruginosa*, *Acinetobacter baumannii*, *Escherichia coli,* and *Stenotrophomonas maltophilia* (4–9).

Here, we report a rare but potentially critical limitation associated with the use of the pUC18T-mini-Tn*7*T system during the expression of a complex, membrane-bound protein. Specifically, we observed that overexpression of the membrane-bound resistance-nodulation-division efflux pump AdeIJK in *A. baumannii* led to a mutation in *lacI^q^*, resulting in constitutive gene expression independent of IPTG.

**Fig 1:**
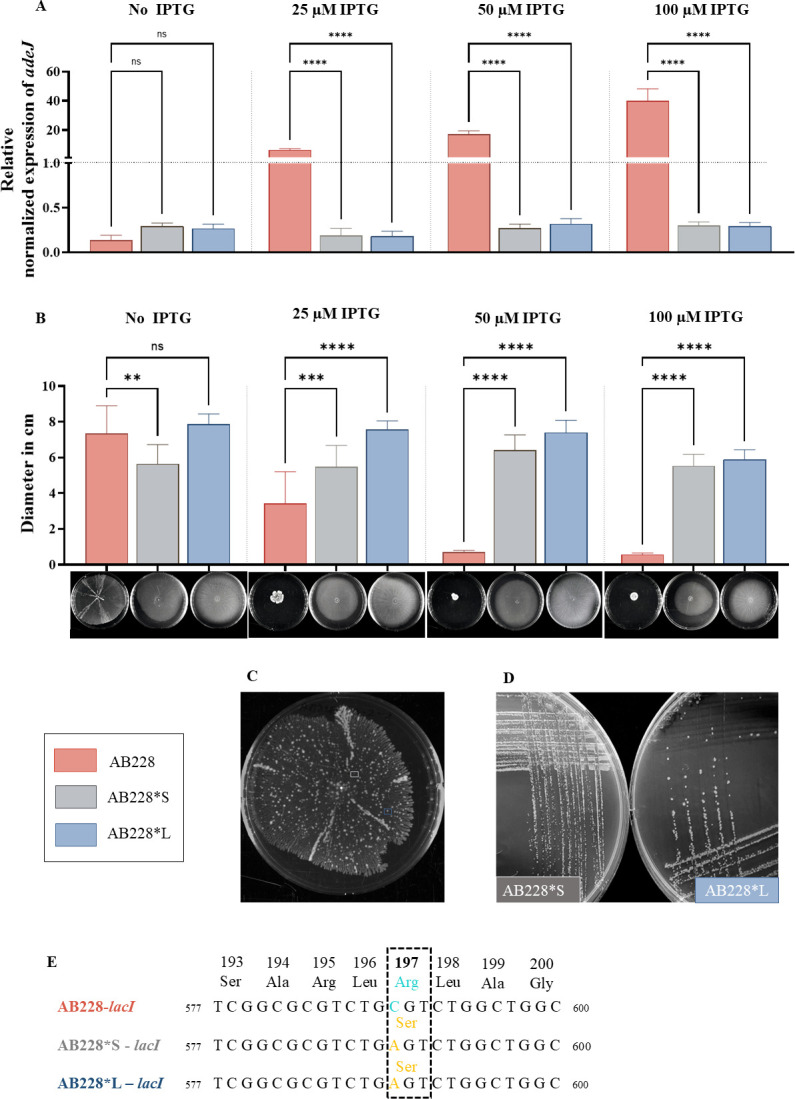
(**A**) Relative induced expression of *adeJ* in *A. baumannii* AB228, AB228*S, and AB228*L when cultured in the presence of increasing concentrations of IPTG. (**B**) Surface-associated motility of standardized AB228, AB228*S, and AB228*L cultures with increasing IPTG concentrations. Motility was determined by measuring the diameter of the culture at three different points on the agarose assay plates after 18 h. Representative images of motility assay plates for each strain are correspondingly displayed below the graph. (**C**) Emergence of distinct colony morphologies (AB288*S, gray box; AB288*L, blue box) displaying a reversed motility phenotype from AB228 (ATCC17978-VU;Δ*adeIJK att*Tn7::*adeIJK*) on motility plates containing 100 µM IPTG after 48 h. (**D**) Small (left; AB228*S) and large (right; AB228*L) colony morphology variants isolated from the motility plate shown in (**C**). (**E**) Multiple sequence alignment of the 577–600 base region of *lacI*, highlighting a single nucleotide difference and the resulting amino acid substitution. Residue numbers are shown above the aligned amino acids. Images in B, C, and D were acquired using an Axygen Gel Documentation System under white light (200 ms exposure). All assays were performed in triplicate in each of three independent experiments. A one-way ANOVA was used for data analysis using GraphPad Prism 10.4: *P* <0.0001 = ****, *P* <0.0002 = ***, *P* <0.0021 = **, *P* <0.0332 = *, 0.1234 = ns (not significant).

Our investigation started with the deletion of the *adeIJK* operon from *A. baumannii* ATCC17978-VU as previously described (10), creating ATCC17978-VU;Δ*adeIJK*. Insertional complementation of *adeIJK* into this deletion strain using a pUC18T-mini-Tn*7*T vector was achieved (2, 11) to generate a new strain that we named AB228 (ATCC17978-VU;Δ*adeIJK att*Tn7::*adeIJK*). The unmarked complemented strain was created by excising the vector-borne gentamicin selection marker flanked by flippase recognition target sites (10). Induction of *adeIJK* in AB228 was verified by RT-qPCR using *adeJ-*specific primers (Table 1) (3). A dose-dependent increase in expression was observed, with 100 µM IPTG resulting in a 40-fold increase relative to uninduced AB228, after normalization to the 16S rRNA housekeeping gene (Fig. 1A**,** AB228).

**TABLE 1:** Primers used in this study

Primer name	Sequence (5′ to 3′)	Use
*adeJ* forward	CATCGGCTGAAACAGTTGAA	Confirmation of inducible expression
*adeJ* reverse	GCCTGACCATTACCAGCACT	
16S forward	CTTCGGACCTTGCGCTAATA	Normalization for RT-qPCR
16S reverse	ATCCTCTCAGACCCGCTACA	
*glmS*	TATGGAAGAAGTTCAGGCTC	Confirmation of the genomic insertion
Tn*7*R	CACAGCATAACTGGACTGATTTC	
*adeIJK*_KO_fwd	ACTGCTTTGGCTCTGGTT	Confirmation of the genomic deletion
*adeIJK*_KO_rev	ACCCGTAAGTTCACCACC	
*ompA*_fwd	CTATGCTTGTTGCTGCTCCA	Confirmation of *A. baumannii* strain
*ompA*_rev	CCATTGTTGTGTTGGCTGTC	
*adeI*_rev	TGCAGTAACCAAAGCACCAG	Used with *glmS* to amplify *lacI* for Nanopore sequencing

AdeIJK has previously been linked with *A. baumannii* motility (12), and we routinely use a surface-associated motility assay with newly created strains as part of their phenotypic assessment. Following a standard protocol and using standardized cultures (10), we noticed that controlled expression of *adeIJK* caused a significant, IPTG-dependent decrease in motility compared to uninduced AB228 after 18 h (Fig. 1B**,** AB228). We continued to monitor motility for 48 h and, surprisingly, observed a notable shift to increased motility in AB228 induced with 100 µM IPTG (Fig. 1C). Distinct-looking colonies observed on this motility plate were streaked onto LB agar revealing the presence of two morphologically different colony types, small and large (Fig. 1D**,** AB228*S and AB228*L). To rule out contamination, we performed PCR (Table 1) on purified genomic DNA from both colony types and confirmed the presence of the *adeIJK* genomic insertion at the *att*Tn*7* site, the initial deletion of the native *adeIJK* operon, and *ompA*, a unique marker for *A. baumannii* (13). The identification of *A. baumannii* was confirmed subsequently by whole genome sequencing (BioProject PRJNA1282226).

We then conducted another motility assay using the isolated small (AB228*S) and large (AB228*L) colony variants. Interestingly, unlike the previous observation, surface-associated motility of both variants did not exhibit a dose-dependent or any response to IPTG after 18 hours (Fig. 1B**,** AB228*S and AB228*L), with AB228*L demonstrating a 12-fold increase in motility in the presence of 100 µM IPTG compared with the first induced AB228 observation under equivalent conditions (Fig. 1B**,** AB228-100 and AB228*L-100). To evaluate whether *adeIJK* expression remained inducible in the variants, we again performed RT-qPCR and found no dose-dependent increase in *adeJ* transcription (Fig. 1A**,** AB228*S and AB228*L), indicating that these mutants were no longer responding to IPTG. To investigate the underlying cause of this observation, we sequenced a 2730 bp PCR fragment from AB228*S and AB228*L (Plasmidsaurus, Nanopore technology) that spanned from the genomic *att*Tn*7* insertion site to *adeI*, which included the *lacI* promoter region. The fastq files were aligned with the original AB228 sequence (whole-genome sequence previously determined using Nanopore technology) using the multiple sequence alignment function in Geneious Prime (https://www.geneious.com/). We identified an arginine-to-serine substitution at position 197 (R197S) of LacI in both AB228*S and AB228*L (Fig. 1E).

LacI is a well-characterized regulatory protein with an N-terminal DNA-binding domain (amino acids 1−60), a core ligand-binding domain (amino acids 61–330), and a C-terminal tetramerization domain (amino acids 331–360) (14). The mutation identified in this study, R197S, lies within the ligand-binding domain. Structural studies suggest that IPTG stabilizes the LacI repressor through hydrogen bonding, with oxygen atoms in the galactose ring of IPTG forming direct links with three LacI amino acid residues, including R197 (14). The R197S substitution, going from a positively charged polar amino acid to an uncharged polar amino acid, may have disrupted this interaction, reducing the binding ability between LacI and IPTG (15). Indeed, previous studies have demonstrated that substitution of R197 with glycine, leucine, or lysine significantly reduced the affinity of IPTG to LacI (15). Furthermore, Kwon et al. (16) reported the emergence of the same R197S mutation in *E. coli* BL21(DE3) during plasmid-based overexpression of a membrane protein (16). They inferred a similar scenario, that the LacI repressor remained bound to the operator sequence with wild-type affinity and no longer responded to IPTG (17). A literature search of mini-Tn7 genomic insertions across 19 different genera showed no published reports describing a *lacI* mutation similar to that presented here, suggesting that our observation was possibly strain or protein-specific but certainly is rare.

To our knowledge, this is the first report of a *lacI* mutation occurring within a single copy genomic insertion in *A. baumannii*. It remains unclear whether this mutation arose spontaneously or as part of a broader cellular stress response. Given the presumed physiological burden associated with consistent expression of a tripartite, membrane-spanning protein, it is plausible that selective pressure favored the emergence of the mutation. We are currently investigating the underlying cause(s) of the observed small and large colony variants. The purpose of this work is to highlight the potential for mutations in the regulatory elements of the mini-Tn*7*-based gene expression system and to stress the importance of closely monitoring regulatory elements when using inducible systems for gene complementation.
